# Supplementary figures and images for: Transcriptome sequencing and analysis reveals the molecular response to selenium stimuli in Pueraria lobata (willd.) Ohwi
Source: PeerJ. 2020 Mar 24;8:e8768. doi: 10.7717/peerj.8768 (PMC7100600; doi:10.7717/peerj.8768)

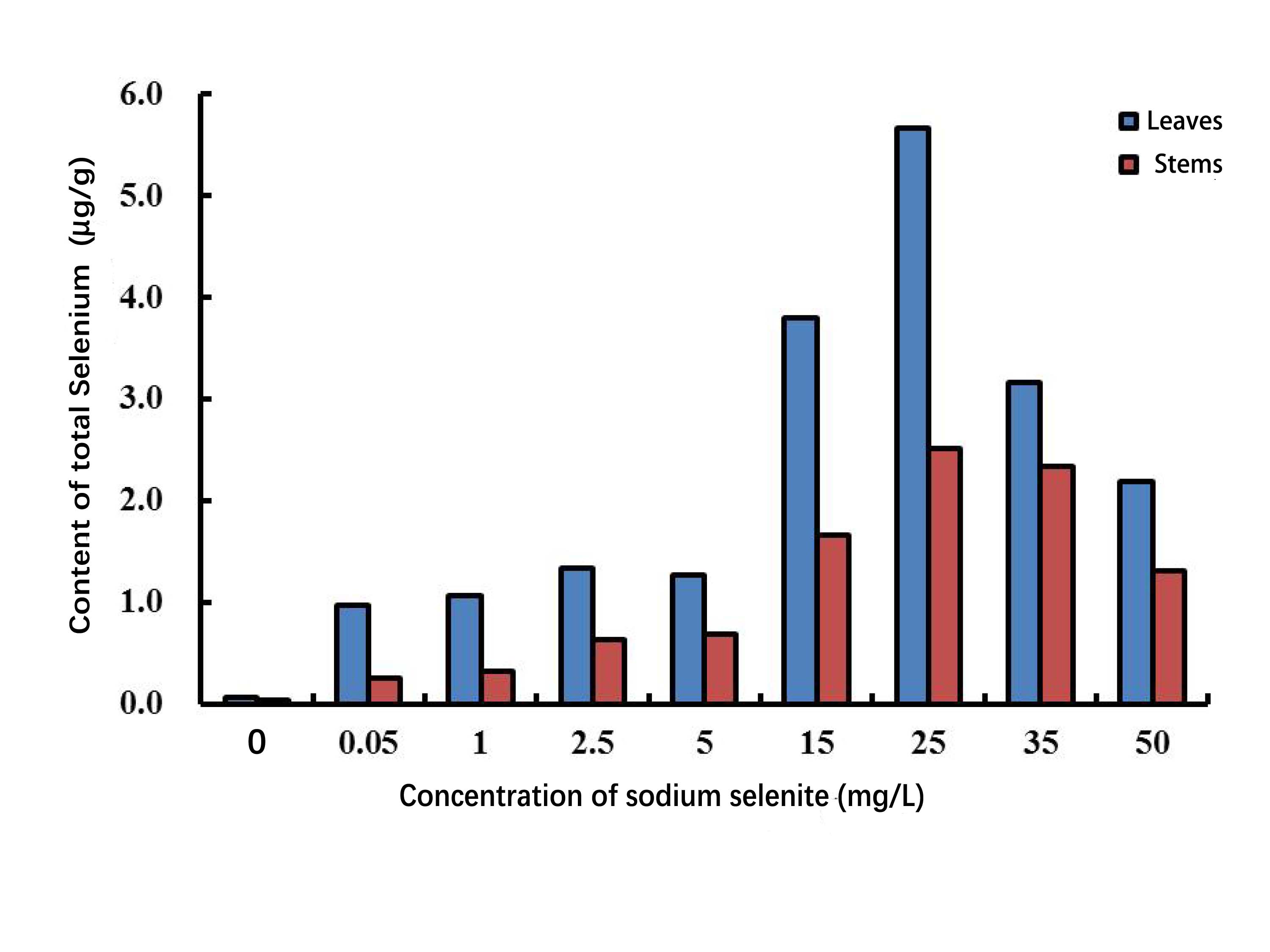

Supplement: Figure S1 [file peerj-08-8768-s001.jpg]

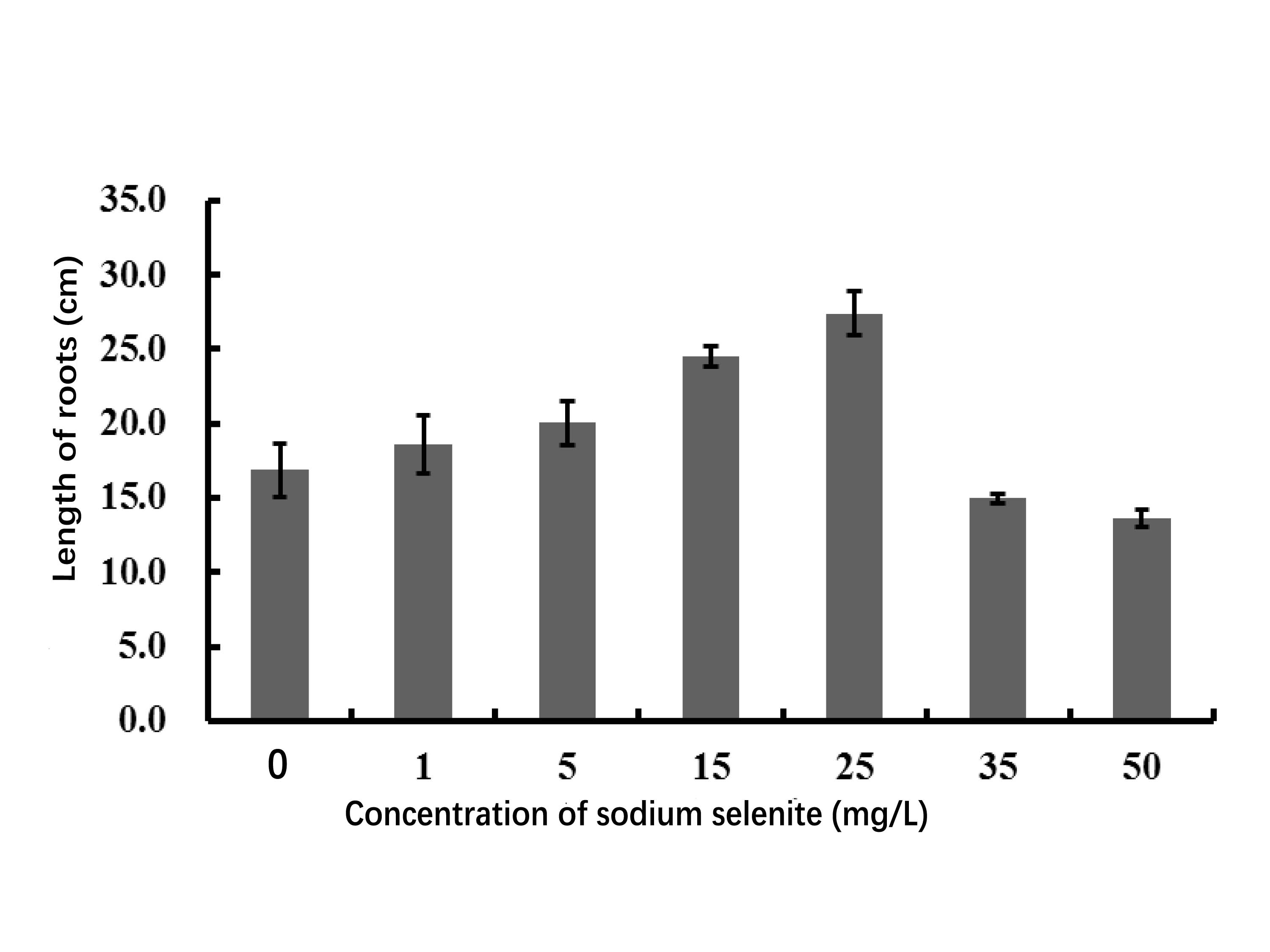

Supplement: Figure S2 [file peerj-08-8768-s002.jpg]

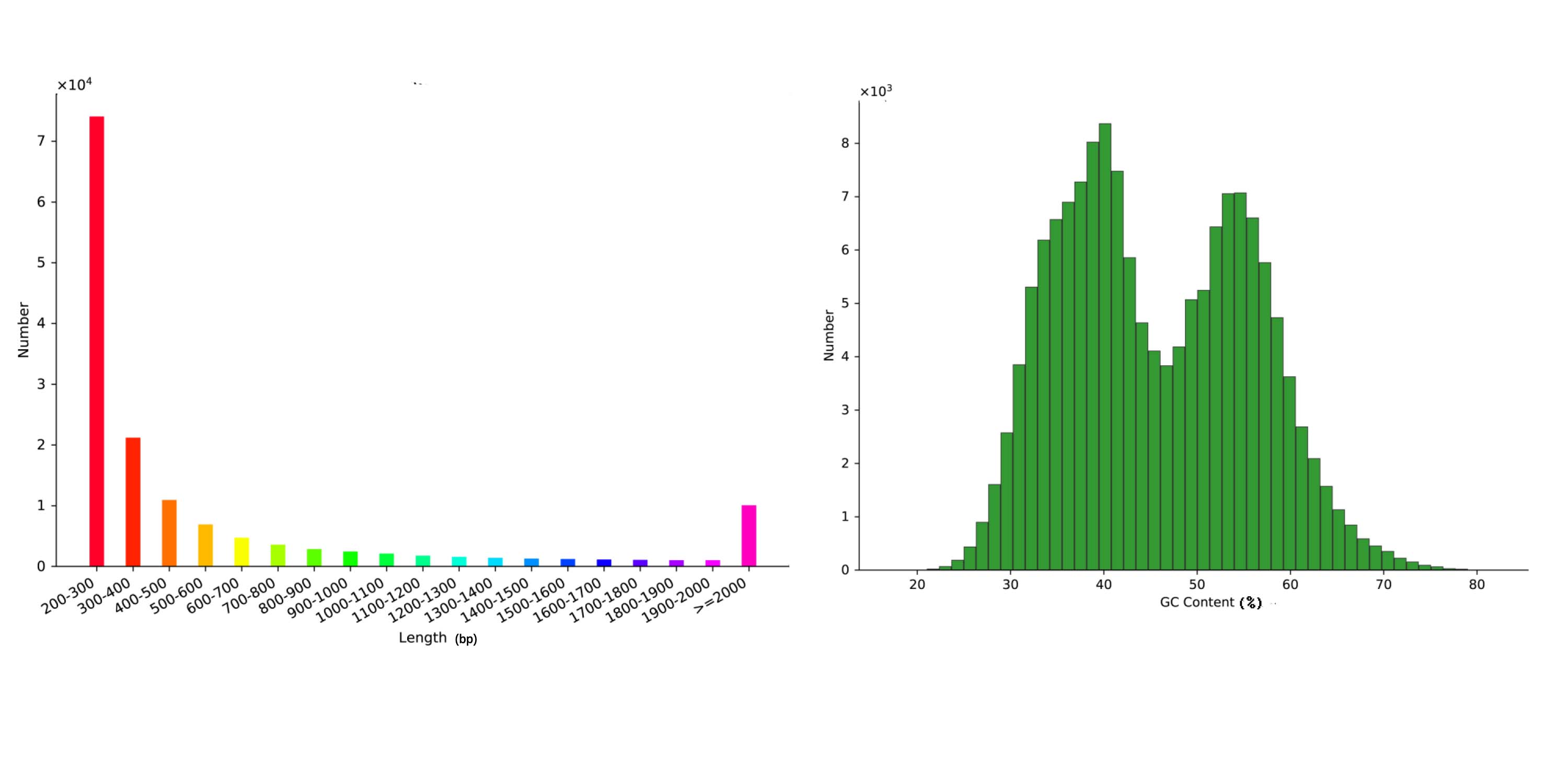

Supplement: Figure S3 [file peerj-08-8768-s003.jpg]

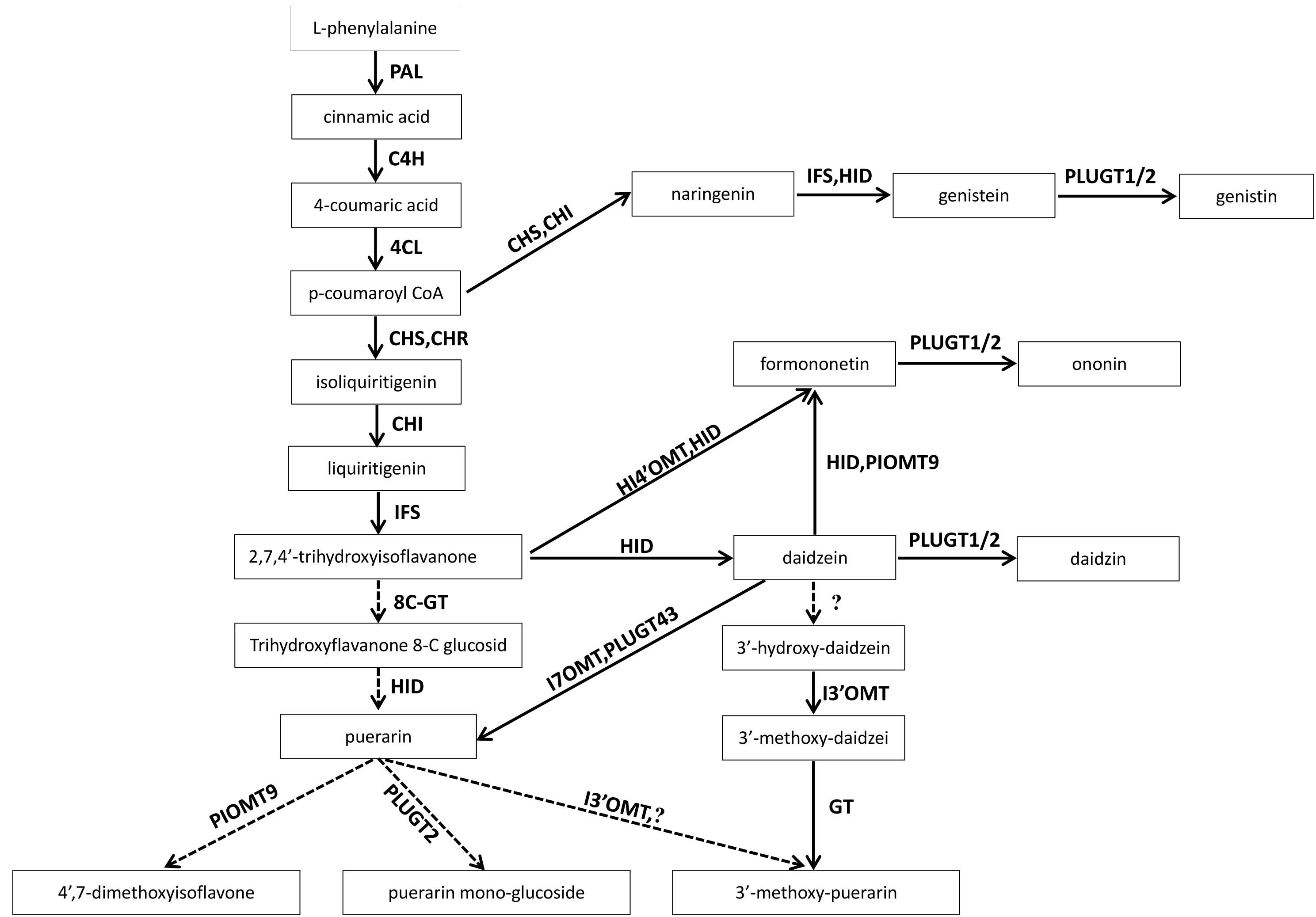

Supplement: Figure S4 — PAL: Phenylalanine Ammonia-lyase ; CA4H: Trans-cinnamate4-monooxygenase; 4CL: 4-CoumaroneCoenzymeALigase; CHS: 6-deoxychalcone synthase; CHI: Chalconeisomerase; IFS: 2-hydroxyisoflavanone synthase; HID: 2-hydroxyisoflavonedehydrates; PLUGT43: PuerariaUDPglucosyltransferase 43; PLUGT1/2: PuerariaUDPglucosyltransferase1/2; I7OMT : isoflavone-7-O-methyltransferase . IF7MAT: isoflavone 7-O-glucoside-6”-O-malonyltransferase; I3 ’ OMT: isoflavone-3’-O-methyltransferase; PIOMT9: isoflavone-4’-O-methyltransferase. [file peerj-08-8768-s004.jpg]

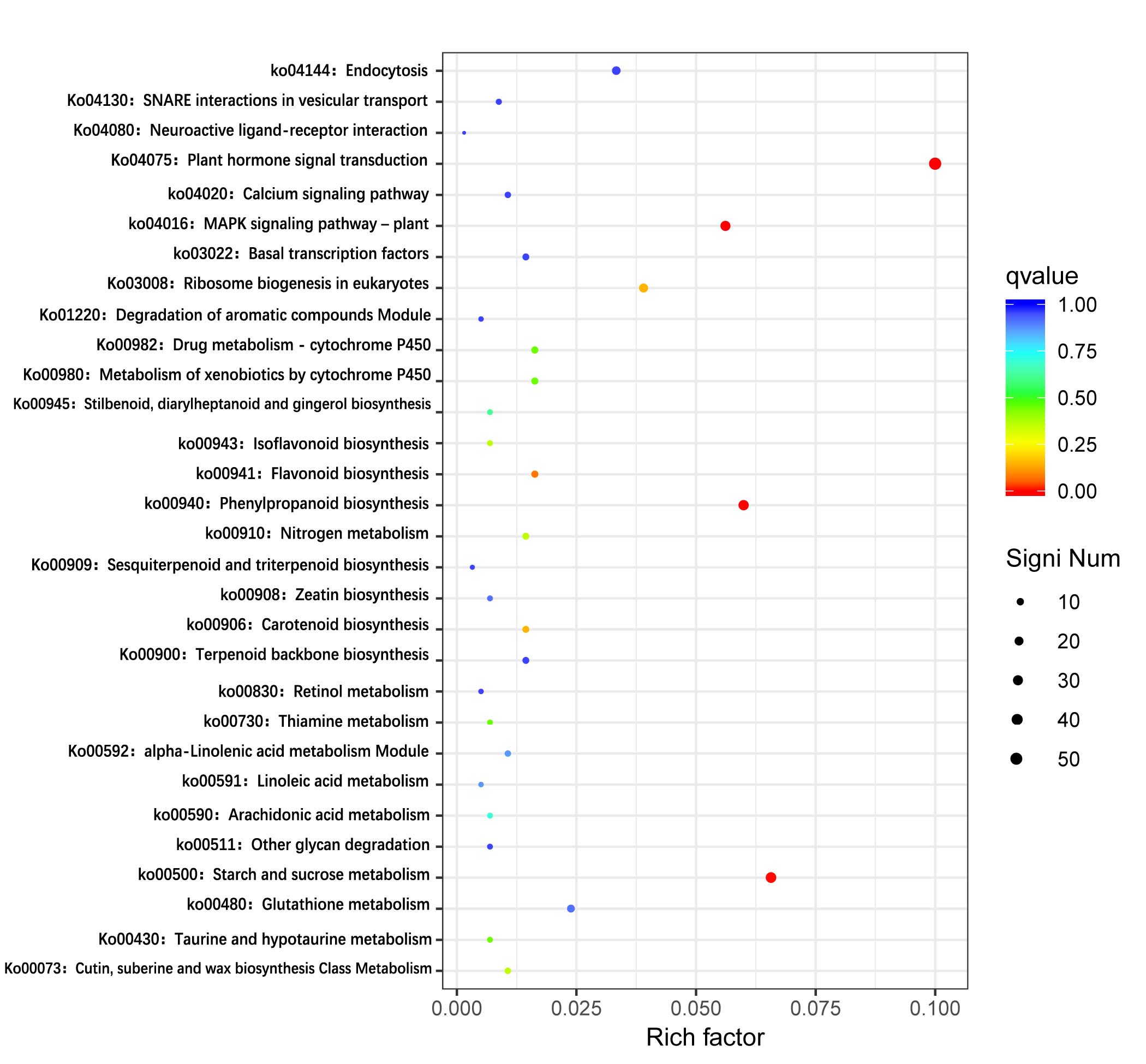

Supplement: Figure S5 — The X-axis represents the enrichment factor and log10 (Q-value). The Y-axis indicates the different KEGG pathways. [file peerj-08-8768-s005.jpg]
