# Supplementary material for: Transcriptome sequencing and analysis reveals the molecular response to selenium stimuli in Pueraria lobata (willd.) Ohwi
Source: PeerJ. 2020 Mar 24;8:e8768. doi: 10.7717/peerj.8768 (PMC7100600; doi:10.7717/peerj.8768)
Supplement: Table S1 [file peerj-08-8768-s006.docx]

**Table S1 Primers used for qRT-PCR**

| **Genes ID** | **Primers** | **Sequences** |
| --- | --- | --- |
| T_43687_c4_g4 | Forward | TGTTTGGTTCTACGGTGGGAGG |
|  | Reverse | AGACCCTGACCATCTCGCAGGT |
| T_43074_c0_g3 | Forward | CAGGGTGTGGCTTGATTCAGTTC |
|  | Reverse | ACAATGGGACCGACAGTTACGG |
| T_40122_c2_g1 | Forward | GTGAAGCCAACCCTCTGAATCTAT |
|  | Reverse | CCGAAACTGGTCGTTCTCTTGG |
| T_41767_c1_g1 | Forward | GGCGATTTCATTCCCATCCTTAGAC |
|  | Reverse | GTTATTGGTGCTCGTGGTGCTTC |
| T_45464_c1_g2 | Forward | GCCACACTACGCAGAACAACAGTC |
|  | Reverse | CTTGGATGAGATGAGGAGGAAATC |
| T_40196_c1_g3 | Forward | CTTGGACCTGTATCTCATCCACTG |
|  | Reverse | GAGAAGTTGCTGACTCCAATGG |
| T_46470_c0_g3 | Forward | GGAATGTCTCCTTCACAATGGCTC |
|  | Reverse | AACAGAGTGGGCTTTGGCAGAG |
| T_41375_c0_g6 | Forward | AAGGTTAGCACCGCTGGTTTCACC |
|  | Reverse | CCCAAGCCAACATAATAGCCGTG |
| T_44141_c2_g2 | Forward | CGAACCACCTTAGGAATGGCAAG |
|  | Reverse | ACGGAGAGGAGAGTGAGATGAAG |
| T_40962_c1_g4 | Forward | CAGTTTGGGATTTGAGTTGGGAAG |
|  | Reverse | GAAGTTGACAGGGTTGTTGGCG |
| PB8162（40S） | Forward | TGAAGCAGTAACTCGCAAGACTC |
|  | Reverse | TGAAAGGAGCAGCATCAACCTGA |
