# Supplementary material for: Transcriptome sequencing and analysis reveals the molecular response to selenium stimuli in Pueraria lobata (willd.) Ohwi
Source: PeerJ. 2020 Mar 24;8:e8768. doi: 10.7717/peerj.8768 (PMC7100600; doi:10.7717/peerj.8768)
Supplement: Table S2 [file peerj-08-8768-s007.doc]

Table S2 Correlation analysis of treatment concentration of sodium selenite and sampling days on SOD activity

|  | SC | Day 0 | Day 1 | Day 3 | Day 5 | Day 7 | Day 9 |
| --- | --- | --- | --- | --- | --- | --- | --- |
| SC | 1 | 0.000 | 0.140 | 0.397 | 0.366 | -0.499^*^ | -0.701^**^ |
| Day 0 |  | 1 | -0.163 | -0.184 | 0.102 | -0.056 | 0.017 |
| Day 1 |  |  | 1 | 0.523^*^ | 0.522^*^ | 0.527^*^ | 0.281 |
| Day 3 |  |  |  | 1 | 0.831^**^ | 0.428 | 0.227 |
| Day 5 |  |  |  |  | 1 | 0.521^*^ | 0.350 |
| Day 7 |  |  |  |  |  | 1 | 0.887^**^ |
| Day 9 |  |  |  |  |  |  | 1 |

Note: * *p*<0.05; ** *p*<0.01. SC: Sample concentration.
