# Supplementary material for: Transcriptome sequencing and analysis reveals the molecular response to selenium stimuli in Pueraria lobata (willd.) Ohwi
Source: PeerJ. 2020 Mar 24;8:e8768. doi: 10.7717/peerj.8768 (PMC7100600; doi:10.7717/peerj.8768)
Supplement: Table S3 [file peerj-08-8768-s008.doc]

Table 3 Correlation analysis of the treatment concentration of sodium selenite and sampling days on MDA content

|  | SC | Day 0 | Day 1 | Day 3 | Day 5 | Day 7 | Day 9 |
| --- | --- | --- | --- | --- | --- | --- | --- |
| SC | 1 | 0.000 | 0.513^*^ | 0.264 | 0.728^**^ | 0.680^**^ | 0.690^**^ |
| Day 0 |  | 1 | 0.426 | -0.002 | 0.014 | -0.039 | -0.071 |
| Day 1 |  |  | 1 | 0.656^**^ | 0.758^**^ | 0.798^**^ | 0.756^**^ |
| Day 3 |  |  |  | 1 | 0.601^**^ | 0.748^**^ | 0.726^**^ |
| Day 5 |  |  |  |  | 1 | 0.910^**^ | 0.929^**^ |
| Day 7 |  |  |  |  |  | 1 | 0.964^**^ |
| Day 9 |  |  |  |  |  |  | 1 |

Note: * *p*<0.05; ** *p*<0.01. SC: Sample concentration.
