# Supplementary material for: Transcriptome sequencing and analysis reveals the molecular response to selenium stimuli in Pueraria lobata (willd.) Ohwi
Source: PeerJ. 2020 Mar 24;8:e8768. doi: 10.7717/peerj.8768 (PMC7100600; doi:10.7717/peerj.8768)
Supplement: Table S4 [file peerj-08-8768-s009.docx]

**Table S4. Statistics of gene annotation**

| Annotated database | Number of genes | Percentage（%） |
| --- | --- | --- |
| CDD | 46,439 | 30.84 |
| KOG | 33,401 | 22.18 |
| NR | 59,266 | 39.36 |
| NT | 69,142 | 45.92 |
| PFAM | 31,796 | 21.12 |
| Swissprot | 58,101 | 38.59 |
| TrEMBL | 58,917 | 39.13 |
| GO | 64,759 | 43.01 |
| KEGG | 5,415 | 3.6 |
| at least one database | 90,961 | 60.41 |
| all database | 3,262 | 2.17 |
| Total genes | 150,567 | 60. 41 |
